# Supplementary figures and images for: First Report of Blood Fluke Pathogens with Potential Risk for Emerging Yellowtail Kingfish (Seriola lalandi) Aquaculture on the Chilean Coast, with Descriptions of Two New Species of Paradeontacylix (Aporocotylidae)
Source: Pathogens. 2021 Jul 6;10(7):849. doi: 10.3390/pathogens10070849 (PMC8308527; doi:10.3390/pathogens10070849)

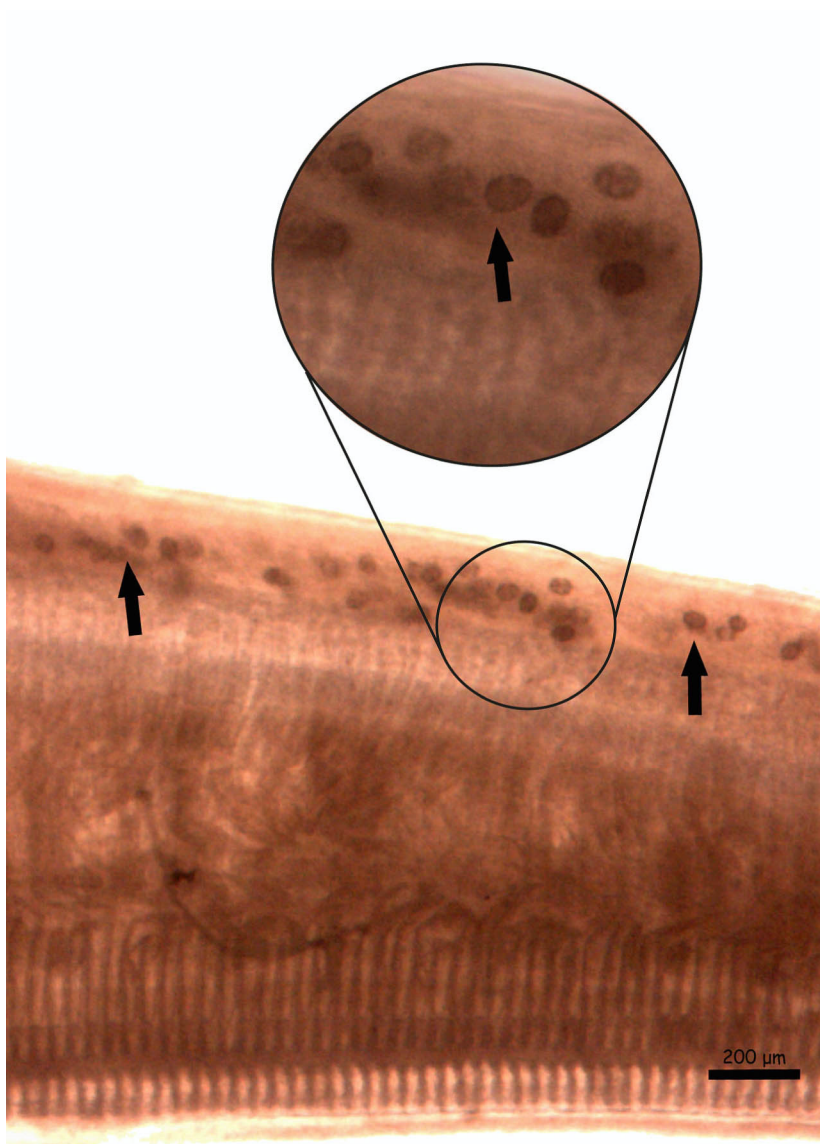

Supplement: Supplementary file 1 [file pathogens-10-00849-s001.zip › Figure S1.pdf]
